# Rapid de-escalation of anti-MRSA therapy guided by *S. aureus* nares screening for patients with pneumonia: protocol of a randomized controlled trial (SNAP study)

**DOI:** 10.3389/fmed.2024.1416904

**Published:** 2024-09-10

**Authors:** Elda De Vita, Francesco Vladimiro Segala, Francesco Cavallin, Giacomo Guido, Luisa Frallonardo, Sergio Cotugno, Carmen Pellegrino, Francesco Di Gennaro, Annalisa Saracino

**Affiliations:** ^1^Clinic of Infectious Diseases, Department of Precision and Regenerative Medicine and Ionian Area (DiMePRe-J), University of Bari Aldo Moro, Bari, Italy; ^2^Independent Statistician, Solagna, Italy

**Keywords:** methicillin-resistant *Staphylococcus aureus*, pneumonia, clinical trial, antimicrobial stewardship, PCR-assay, protocol

## Abstract

**Introduction:**

The current Infectious Disease Society of America and American Thoracic Society (IDSA/ATS) guidelines recommend linezolid or vancomycin as an empiric treatment for methicillin-resistant *Staphylococcus aureus* (MRSA) pneumonia in hospitalized patients with specific risk factors,. A nasal PCR-assay for MRSA, with its high negative predictive value, can guide a rapid antibiotic de-escalation avoiding unnecessary anti-MRSA treatment. The indiscriminate use of these drugs has contributed to the emergence of resistant *S. aureus* strains leading to adverse effects without any survival benefit, increasing hospital stays and associated costs. Aim of the study is the use of this diagnostic tool to reduce empirical anti-MRSA treatment duration in pneumonia, shortening antimicrobial therapy days while measuring in-hospital mortality, length of stay and adverse drug event incidence.

**Methods:**

It is a prospective, randomized single-center controlled trial planned to be conducted in the Azienda Consorziale Policlinico di Bari. The research project will have a duration of 12 months following the approval of the Ethical Committee of the University of Bari. The minimum sample size is 38 patients per group, for a total of 76 subjects, calculated assuming a standard deviation of 10, a power of 90%, a type I error of 5% and a 10% drop-out rate. We will enroll eligible patients ensuring their evidence-based management according to guidelines, we will perform a nasal swab for MRSA in patients in the experimental group and discontinue the empirical anti-MRSA therapy if the nasal swab result is negative. For both arms, follow-up visits will be on day 2, 5, 7, 14, and 28 relatives to the enrollment visit (day 0). Data will be collected on the clinical course of pneumonia and laboratory tests.

**Discussion:**

Our study will provide evidence on the duration (in days) of the antibiotic intake as a primary outcome of the study. Secondary outcome measures include in-hospital mortality, the length of stay and days of mechanical ventilation (in VAP), and the incidence of adverse events related to the administration of the therapy.

**Clinical trial registration:**

https://classic.clinicaltrials.gov/ct2/show/NCT06238297, identifier NCT06238297.

## Introduction

Based on current Infectious Disease Society of America and American Thoracic Society (IDSA/ATS) guidelines, antimicrobial agents such as linezolid and vancomycin are commonly used in hospitalized patients in settings with high community methicillin-resistant *Staphylococcus aureus* (MRSA) rate as an empirical treatment in patients with pneumonia (1). In particular the empirical use of these agents finds application in community acquired pneumonias (CAPs), including the former category of healthcare associated pneumonia (HCAP), as well as in hospital-acquired pneumonias (HAPs) pneumonia occurring after 72 h of hospital admission and ventilator associated pneumonias (VAPs) occuring after 72 h of mechanical ventilation, suggesting the empiric use of such anti-MRSA therapy for patients with specific risk factors (e.g., chronic lung disease, pre-admission wound care, non-ambulatory, diabetes, hospitalization in the past 90 days, chronic hemodialysis, exposure to parenteral antibiotics and/or previous isolation of these organisms, in particular from the respiratory tract) while obtaining culture data (1).

According to the 2023 annual report of the European Antibiotic Resistance Surveillance System (EARSS) there is a high prevalence of MRSA throughout the European territory (2), however, even in countries with high endemicity, the presence of MRSA has remained stable in the last years (3). In Italy, according to the National Antibiotic-Resistance Surveillance System (AR-ISS), in 2021 the percentage of blood isolates MRSA isolates showed a reduction (29.9%) in the national territory, standing, however, at 33.6% in the Puglia region, a percentage higher than the European (4).

In patients with pneumonia, the nasal PCR-assay for MRSA has a high negative predictive value (NPV) (5), with the crucial implication that a negative result can be used to guide the antibiotic de-escalation, avoiding an improper empiric use of the anti-MRSA therapy (6). Data supporting the clinical validity of the use of the nasal swab for MRSA as a diagnostic tool for pneumonia are robust, as demonstrated by many retrospective studies, one of them conducted by Mergenhagen et al., in a cohort of 245,000 unique patients on more than 500,000 clinical cultures from various anatomical sites, in which the NPV of MRSA nares screening to rule out MRSA infection was of 96.5% (7) for any infections and 98.6% for pneumonia. According to Parente et al. (6), the sensitivity and specificity of MRSA nares screen in pneumonia were 70.9 and 90.3%, respectively, with higher values for CAP/HCAP, 85 and 92.1%, respectively (6). A more recent Italian study of 2022 by Colaneri et al. (8) conducted on 1,461 patients with VAP demonstrated a low sensitivity of MRSA nasal-swab testing with a specificity of 98.4% and a NPV of 98.7%. Furthermore, the aforementioned study reported a decreased prevalence in MRSA VAP over the last 10 years (from 9.4% in 2012 to 1.3% in 2021), while methicillin-susceptible *Staphylococcus aureus* (MSSA) remained steady (from 12.3% in 2021 to 7.1% in 2021) (8). These findings have been confirmed by prospective studies such as the one conducted by Paonessa et al., demonstrating that the use of a rapid PCR test for MRSA on Broncho-Alveolar Lavage (BAL) samples significantly reduced the use of anti-MRSA therapy in ventilated patients with suspected pneumonia decreasing incidence of adverse effects a reducing in-hospital mortality (9).

In this regard, as stated by the IDSA/ATS guidelines, anti-MRSA treatment can be avoided in case of negative result, especially in non-severe CAP, thus preventing the misuse of anti-MRSA drugs (7).

The indiscriminate use of these drugs over time has considerably led to the selection of resistant strains of *S. aureus* such as Vancomycin-intermediate *Staphylococcus aureus* (VISA), heterogeneous VISA (hVISA) and Vancomycin-resistant *Staphylococcus aureus* (VRSA) (10). Furthermore, the high risk of adverse events given by the empirical use of anti-MRSA drugs should also be noted including, among others, nephrotoxicity and ototoxicity with vancomycin and bone marrow suppression and peripheral neuropathy with linezolid, without a gain in terms of survival and with longer hospitalization stays (11). The de-escalation approach is aimed at shortening the MRSA therapy by approximately 2 days and reduce serum level of vancomycin, as stated by a recent study (12) with no significative difference in clinical outcomes.

In addition, the systematic use of such drugs is associated with an increase in hospital costs, where the PCR assay for MRSA has a relevant role as a diagnostic tool with a relative minimal cost. According to a study conducted by the Stanford University in 2021 (13), enabling antibiotic de-escalation within 24 h, its use has of facts led to a cost saving of vancomycin of $40,33 per patient, while another American study observed a cost reduction for vancomycin of $108 per patient (14).

On these assumptions, it is clear that using the nasal swab for MRSA in pneumonia diagnosis avoids the misuse of drugs; reducing the risk of iatrogenic toxicity, hospital stay days and the duration of ventilation in VAPs, giving a large impact on health costs.

## Methods and analysis

### Objectives

The aim of our study is to implement the use of the nasal swab for MRSA as a diagnostic tool reducing the duration of the empirical anti-MRSA treatment in the population of hospitalized patients with pneumonia.

The main endpoint of the study will be to evaluate the duration in terms of days of the antimicrobial therapy, while secondary endpoints will be the estimation of intrahospital mortality, length of stay and days of mechanical ventilation (in VAPs). Additionally, we will measure the incidence of adverse events related to the administration of empirical anti-MRSA antibiotic therapy and the hospital costs.

### Design of the study

It is a prospective, randomized single-center controlled trial conducted in the Azienda Consorziale Policlinico di Bari.

#### Study population

Enrollment of all subjects admitted in medical unites with radiological and clinical signs of pneumonia on treatment with anti-MRSA drugs within 48 h, aged 18 or older, regardless of gender and nationality. They will be included in the study provided that informed consent is signed and that the inclusion criteria are met.

The inclusion criteria are:

• Subjects 18 years or older;
• Patients hospitalized at the Azienda Consorziale Policlinico di Bari;
• Clinical diagnosis of CAP/HAP/VAP including radiological and clinical signs and symptoms of pneumonia based on clinical judgment of the researcher responsible for enrollment together with the prescribing physician;
• Commitment by the prescribing physician to set an anti-MRSA antibiotic therapy in empirical;
• Enrollment within 48 h from the beginning of the empirical anti-MRSA therapy.

While the exclusion criteria are:

• Febrile neutropenia or severe immunodeficiency;
• Chronic airway infection (e.g., cystic fibrosis, tuberculosis, aspergillosis);
• Suspect of extrapulmonary infection by MRSA;
• Refusal by the patient or legal guardian;
• Refusal by the physician in charge of the patient to perform antibiotic de-escalation based on the result of the nasal swab;
• Enrollment after 48 h from the beginning of the empirical anti-MRSA therapy.

The study will involve two phases: a first phase of enrollment of the subjects, which includes the collection of informed consent, and of the anamnestic and personal data.

Potential difficulties in this phase might be similar to results obtained in other trials, in particular linked to the refusal by the physician in charge to perform antibiotic de-escalation or more than 48 h of MRSA treatment prior to enrollment (9).

#### Randomization

After obtaining informed consent, each participant will be randomly assigned to the experimental or control group. Randomization will be performed using a computer-generated random assignment list and will be balanced according to sex and age with parallel assignment. Participants in the experimental group will carry out a swab within 48 h of starting empirical anti-MRSA therapy, which will be suspended in the event of a negative swab result.

Participants in the control group will continue empirical anti-MRSA therapy as per usual procedure (Supplementary Table 1).

#### Procedures

The implementation of these steps will be ensured by the performance of the tasks foreseen for each unit (Table 1). Each clinical unit will:

– Enroll patients and collect demographic, clinical, and biochemical data at baseline, as well as cultural samples (sputum culture, nasal swab culture and hemoculture), ensuring their management according to guidelines and scientific evidence for the entire duration of the follow-up.
– Perform a nasal swab for MRSA in patients in the experimental group and discontinue the empirical anti-MRSA therapy if the nasal swab result is negative. If the MRSA screening test returns a positive result, primary care teams will be advised to adhere established clinical guidelines for MRSA management. This will include confirming the result with cultural tests, evaluating the patient for possible decolonization protocols, and implementing appropriate infection control measures to prevent the spread of MRSA.
– Continue therapy in the control group, the eventual de-escalation of anti-MRSA therapy will be performed by the physician based on culture results and clinical judgment. For both arms, follow-up visits will be on day 2, 5, 7, 14, and 28 relatives to the enrollment visit (day 0). At the control visits, data will be collected on the clinical course of pneumonia and laboratory tests. After discharge, the visit can also be conducted by telephone.

**TABLE 1:** Table of the trial procedure timeline.

	Day 0	Day 2	Day 5	Day 7	Day 14	Day 28
Check eligibility	X					
Recruitment	X					
Randomize	X					
Demographics	X					
Microbiological data	X					
Therapy data	X	X	X	X	X	X
Clinical data	X	X	X	X	X	X
Laboratory data	X	+/−	+/−	X		
Vital status assessment	X	X	X	X	X	X

Time path of the enrollment, interventions, assessments, and visits of the participants.

Outcomes will be assessed by the day 28: the primary outcome measure will be the duration (in days) of anti-MRSA therapy. Secondary outcome measures include in-hospital mortality, the length of stay and days of mechanical ventilation (in VAP), and the incidence of adverse events related to the administration of therapy and hospital costs.

#### Timeline

The research project will have a duration of 12 months following the approval of the Ethics Committee of the University of Bari.

### Statistical analysis

The sample size calculation is based on the main outcome measure (duration of antibiotic intake), which we assume to have an average of 10 days in the control group and 2 days in the experimental group. Assuming a standard deviation of 10, a power of 90%, and a type I error of 5%, a minimum of 34 patients per group (thus a total of 68 patients) are required to demonstrate a reduction from 10 to 2 days in the duration of antibiotic intake. To this is added an estimated 10% of cases lost to follow-up, resulting in a final sample size of 76 patients. An interim analysis will be performed when half of the participants will be enrolled. The interim analysis will only verify the initial assumptions about the magnitude of the duration of antibiotic intake in the experimental and control groups, and no statistical testing will be performed. The figures from the interim analysis will inform a possible adaptation of the sample size.

The statistical analysis will be conducted according to intention-to-treat approach. In the unlikely case of contamination between the two treatment groups, a per-protocol analysis will also be performed and the conclusions of the study will consider the results of both approaches. The numerical data will be summarized as mean and standard deviation, and categorical data as absolute and relative frequencies. Group comparisons will be conducted using the Student’s t-test for numerical data and the Chi-Square test or Fisher’s test for categorical data. The effects sizes will be reported as mean differences or relative risks, with 95% confidence intervals. All statistical tests will be two-tailed, and a *p* < 0.05 will be considered statistically significant. The statistical analysis will be performed using R 4.3 (R Foundation for Statistical Computing, Vienna, Austria).
