# Supplementary material for: Rapid de-escalation of anti-MRSA therapy guided by S. aureus nares screening for patients with pneumonia: protocol of a randomized controlled trial (SNAP study)
Source: Front Med (Lausanne). 2024 Sep 10;11:1416904. doi: 10.3389/fmed.2024.1416904 (PMC11420041; doi:10.3389/fmed.2024.1416904)
Supplement: Supplementary file 1 [file Table_1.DOCX]

| Allocation: |  |  |  |
| --- | --- | --- | --- |
| Sequence generation | 16a | Method of generating the allocation sequence (eg, computer-generated random numbers), and list of any factors for stratification. To reduce predictability of a random sequence, details of any planned restriction (eg, blocking) should be provided in a separate document that is unavailable to those who enrol participants or assign interventions | The allocation sequence will be created using a computer-generated random assignment list. The list will be stratified by sex and age, using block randomisation with block sizes of 2 to 4. |
| Allocation concealment mechanism | 16b | Mechanism of implementing the allocation sequence (eg, central telephone; sequentially numbered, opaque, sealed envelopes), describing any steps to conceal the sequence until interventions are assigned | Arm allocations will be put in sequentially numbered, sealed, opaque envelopes. |
| Implementation | 16c | Who will generate the allocation sequence, who will enrol participants, and who will assign participants to interventions | The study statistician will generate the allocation sequence; the research clinicians will enrol the participants; the principal investigator will assign participants to the interventions. |
| Blinding (masking) | 17a | Who will be blinded after assignment to interventions (eg, trial participants, care providers, outcome assessors, data analysts), and how | The participants and the outcome assessors will not be masked because of the characteristics of the interventions. However, the statistician will be masked to treatment allocation. |
|  | 17b | If blinded, circumstances under which unblinding is permissible, and procedure for revealing a participant’s allocated intervention during the trial | N/A |

Additional file 1

Spirit checklist of the study protocol
